# Cardiovascular risk factors in children and adolescents with congenital adrenal hyperplasia due to 21-hydroxylase deficiency

**DOI:** 10.1111/cen.12265

**Published:** 2013-07-08

**Authors:** Anbezhil Subbarayan, Mehul T Dattani, Catherine J Peters, Peter C Hindmarsh

**Affiliations:** * Department of Endocrinology, Great Ormond Street Hospital for ChildrenLondon, UK; † Developmental Endocrinology Research Group, UCL Institute of Child HealthLondon, UK

## Abstract

**Objective:**

The prevalence of cardiovascular risk factors in congenital adrenal hyperplasia (CAH) varies widely. In the light of recent changes in treatment regimens, we have reassessed the prevalence of these risk factors in our current cohort of patients with CAH due to P450c21 deficiency.

**Methods:**

A retrospective cross-sectional study of 107 children (39 m) with CAH aged 9·2 years (range 0·4–20·5 years). Anthropometric, systolic (SBP) and diastolic (DBP) blood pressure data were collected and expressed as standard deviation scores (SDS) using UK growth reference data and the Fourth Task Force data set, respectively. Fasting blood glucose with plasma insulin and lipids was measured, and insulin resistance (HOMA IR) calculated using the homoeostasis assessment model.

**Results:**

23·6% (33% men; 18% women) of the cohort were obese (BMI SDS>2). BMI SDS was significantly higher (*P* < 0·001) when compared with the UK population. Nineteen (20·9%) of 91 patients (20% men; 21% women) had systolic hypertension and 8 [8·8% (8·6% men; 8·9% women)] had diastolic hypertension. Mean SBP [108 (SD 13·5)] mm Hg was significantly higher than the normal population (*P* < 0·001), but mean DBP was not (*P* = 0·07). Both SBP SDS and DBP SDS were not related to BMI SDS. 9·5% of the subjects had hyperlipidaemia, but HOMA IR was more favourable compared with the normal population.

**Conclusion:**

Despite a reduction in steroid doses over the last decade, a number of children with CAH are still obese and hypertensive. Whether this reflects general population trends or indicates a need to further optimize treatment regimens remains to be determined.

## Introduction

Congenital adrenal hyperplasia (CAH) is an autosomal recessive disorder of adrenal steroid biosynthesis most commonly caused by mutations or deletions in the *CYP21A2* gene.^1^ This leads to a variable deficiency of the enzyme 21-hydroxylase (P450c21) leading to a range of manifestations [salt-wasting type (SW CAH), simple virilizing type (SV CAH), nonclassic type (NC CAH)]. Deficiency of P450c21 leads to decreased secretion of cortisol and aldosterone with an increase in steroid precursors prior to the block. Reduced cortisol stimulates adrenocorticotrophic hormone secretion via negative feedback which then leads to adrenal hyperplasia and increased adrenal androgen production.^2^ These individuals also have decreased adrenal medullary function^3^ which has been suggested to lead to obesity and insulin resistance (IR) via the leptin pathway.^4^ The treatment aim in CAH is to replace the deficient hormones (glucocorticoid ± mineralocorticoid) and reduce the excessive androgens. The management of CAH poses a great challenge as the therapeutic window for glucocorticoids is narrow. Current treatment regimens do not match the circadian rhythm often leading to poor control of the disease despite the use of supraphysiological doses.^5^,^6^

The combination of hyper/hypocortisolism, hyper/hypoandrogenism and adrenal medullary hypofunction due to the disease and its treatment may make these individuals more prone to develop cardiovascular risk factors such as obesity, hypertension, IR and dyslipidaemia.^7^ Although treatment has changed, the optimum method of delivery of steroids and monitoring of treatment remains to be defined. Even with the recent publication of guidelines,^8^ there are variations in dosage schedules and methods of monitoring treatment across the globe. This may explain the different prevalence rates reported for cardiovascular risk factors in children and adults.^9^–^15^ Over the last 10 years, we have modified our treatment regimen for P450c21 deficiency in terms of glucocorticoid and mineralocorticoid dosing and have undertaken an audit of practice to reassess the prevalence of these cardiovascular risk factors in our current cohort of patients with CAH due to P450c21 deficiency.

## Patients and methods

### Patients

We undertook a retrospective analysis of the medical records of all patients with CAH due to 21-hydroxylase deficiency who were followed up in the Paediatric Endocrinology Department at Great Ormond Street Hospital for Children between 2007 and 2012. The diagnosis of CAH P450c21 deficiency was based on clinical and biochemical assessment and confirmed in all cases by genetic analysis of the *CYP21A2* gene. All patients were treated with oral hydrocortisone (10–15 mg/m^2^/day) in three or four divided doses. SW CAH was diagnosed in the presence of hyponatraemia, hyperkalaemia, raised plasma renin activity (PRA) and low plasma aldosterone concentrations. In addition to hydrocortisone, these children received 9-alpha-fludrocortisone (50–100 μg/m^2^/day) in one to two divided doses. Infants with SW CAH also received sodium supplements (15–30 mEq/kg/day) in four divided doses until their first birthday. In addition to regular clinic review 3–6 monthly, all underwent annual reviews.

### Methods

All patients with CAH had at least one detailed review per year. Patients were admitted for 24 h. An intravenous cannula was inserted, and plasma cortisol and 17-hydroxyprogesterone (17-OHP) concentrations were measured as detailed in Table S1. At 08·00 h, a further sample was drawn for the measurement of fasting blood glucose and plasma insulin, cholesterol and triglyceride concentrations as well as plasma renin activity (PRA), androstenedione (A4) and testosterone. Six hourly blood pressure measurements were obtained using an oscillometric method (Philips IntelliVue MP30, Germany) with appropriate size cuffs. The average systolic (SBP) and diastolic blood pressures (DBP) were derived from these measurements. Height was measured using a Harpenden stadiometer (Holtain, Crymych, UK) and weight using Seca digital weighing scale (Seca, Birmingham, UK). Body mass index (BMI) was calculated using Quetelet index (weight in kilograms/height in m^2^).

### Hormone assays

#### Cortisol

Serum total cortisol was measured using competitive chemiluminescent immunoassay (Immulite 2000 Siemens Diagnostics). The sensitivity of the assay is 5·5 nmol/l, and the coefficients of variation (CV) were 11% and 4% at the levels 81·9 nmol/l and 499 nmol/l, respectively.

Plasma renin activity (PRA) was measured as described by Menard & Catt.^16^ The assay sensitivity is 0·17 nmol/l/h, and the CV is 4·9% at 0·49 nmol/l/h, 6·2% at 0·84 nmol/l/h and 7·9% at 1·37 nmol/l/h.

Cholesterol was measured using dry slide enzymatic (cholesterol oxidase) method (Vitros 5600 Clinical Chemistry analyser, Ortho Clinical Diagnostics). The sensitivity is 1·29 mmol/l with a CV of 1·8% at 3·8 mmol/l. Triglycerides were measured using this same method with the sensitivity of 0·11 mmol/l and a CV of 1·4% at 1·35 mmol/l.

Glucose was measured using dry slide enzymatic (glucose oxidase) method (Vitros 5600 Clinical Chemistry analyser, Ortho Clinical Diagnostics). The assay sensitivity is 1·1 mmol/l, and the CV is 1·5% at 4·6 mmol/l.

Insulin was measured using a two-site immunometric chemiluminescent immunoassay (Immulite 2000 Siemens Diagnostics). The sensitivity is 2 mU/l with a CV of 17·7% at 3·9 mU/l and 3·9% at 18·9 mU/l.

### Statistics

Anthropometric data were expressed as standard deviation scores (SDS) using the 1990 UK growth reference data.^17^ Patients with BMI SDS >2 were classified as obese.^18^ The systolic and diastolic blood pressures were expressed as percentiles and SDS using the Fourth Task Force data set.^19^ For children <1 year old, the normative values were taken from the Second Task Force report.^20^ Patients with average SBP and/or DBP ≥95th percentile for gender, age and height were classified as hypertensive and those between 90th and 95th centiles as prehypertensive.^19^ IR was calculated using the homoeostasis model assessment (HOMA) method and compared with a data set from 94 short and tall normal individuals at Great Ormond Street Hospital for Children. Fasting total plasma cholesterol concentrations >5·5 mmol/l and fasting plasma triglyceride concentrations >1·55 mmol/l were considered to be elevated.

Statistical analysis was performed using SPSS version 21. Non-normally distributed data were logarithmically transformed prior to analysis. Normally distributed data are expressed as mean and SD, whereas non-normally distributed data were expressed as median and interquartile range (IQR). Mean data were compared between groups using Student’s *t*-test. When more than two groups were compared, one-way analysis of variance (anova) with Tukey’s *post hoc* test was used to determine significance. Stepwise multiple linear regression analysis was used to explore relationships between blood pressure and chronological age, steroid doses, BMI SDS and mean cortisol concentration. A *P*-value <0·05 was considered as depicting significance.

## Results

### General characteristics

The records of 107 patients (39 men), aged 0·4–20·5 years (median 9·2 years) were analysed. Of these, 57 (53·3%) patients were in the age group 0–9·9 years, 37 (34·6%) in the age group 10–14·9 years and 13 (12·1%) were in the age group ≥15 years. Seventy-nine (74%) patients had SW CAH, and the remainder had SV CAH. Table 1 shows the biochemical parameters of control in the patients. With the current regimens, the overall biochemical control was inadequate with high premorning dose 17-OHP concentrations and increased mean 24 h 17-OHP concentrations. Forty-four (63%) of 70 patients had high plasma androstenedione concentrations, and only 23 (33%) had normal levels and 3 (4%) had low levels. Mean PRA was <10 pmol/ml/h, suggesting adequate mineralocorticoid replacement.

**Table 1:** Steroid doses and biochemical markers of control

Age groups	Hydrocortisone (mg/m^2^) Mean (SD)	Fludrocortisone (μg/m^2^) Mean (SD)	Mean 17-OHP (nmol/l) Median (IQR)	Mean cortisol (nmol/l) Median (IQR)	Androstenedione (μmol/l) Median (IQR)	Plasma renin activity (nmol/h/l) Median (IQR)
0–9·9 years	13·3 (4·9)	121·4 (51·5)	*n* = 54	*n* = 55	*n* = 32	*n* = 50
			27 (6–80)	187 (151–214)	2·7 (1–12·7)	5·7 (2·7–9·4)
10–14·9 years	13 (3·5)	84 (39·7)	*n* = 32	*n* = 35	*n* = 28	*n* = 27
			56 (18–132)	175 (144–200)	13·9 (6·8–28·8)	3·9 (2·3–6·5)
≥15 years	14 (4·5)	67 (27·7)	*n* = 13	*n* = 11	*n* = 10	*n* = 11
			61 (9–149)	203 (154–223)	15·9 (5·2–21·5)	3·6 (2–5·7)
All age	13·3 (4·4)	102 (50)	*n* = 99	*n* = 101	*n* = 70	*n* = 88
			38 (8–123)	184 (151–210)	9·3 (2–19)	5 (2·4–7·2)

### Steroid doses

**Figure 1:**
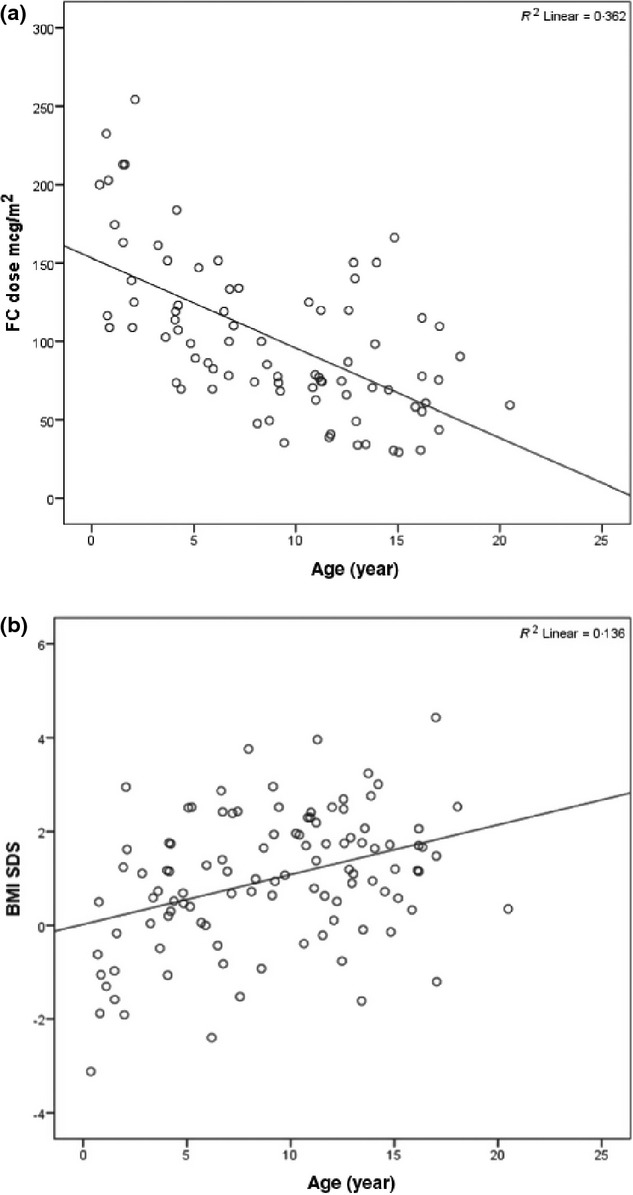
(a) Correlation between chronological age and 9-alpha-fludrocortisone dose. (b) Correlation between age and BMI SDS.

Mean daily hydrocortisone and 9-alpha-fludrocortisone replacement doses were 13·3 (SD 4·4) mg/m^2^ body surface area (BSA) and 102 (SD 50) μg/m^2^ BSA, respectively. These doses varied depending upon age: mean hydrocortisone and 9-alpha-fludrocortisone doses were 13·3 (SD 4·9) mg/m^2^ BSA and 121·4 (SD 51·5) μg/m^2^ BSA, respectively, in the 0-9·9 years age group, 13 (SD 3·5) mg/m^2^ BSA and 84 (SD 39·7) μg/m^2^ BSA, respectively, in the 10–14·9 years age group and 14 (SD 4·5) mg/m^2^ BSA and 67 (SD 27·7) μg/m^2^ BSA, respectively, in the ≥15 years age group (Table 1). There was no relationship between hydrocortisone dose and age, whereas the dose of 9-alpha-fludrocortisone was negatively correlated with age (*r* = −0·6; *P* = 0·000) in both sexes [Fig. 1(a)].

### Obesity

Table 2 shows the anthropometric data in 106 patients in the different age groups. Twenty-five (23·6%) of 106 subjects (33% of men, 17·8% of women; *χ*^2^ = 0·07, *P* = 0·09) in the cohort were obese. In the whole study group, weight SDS and BMI SDS were significantly higher (*P* < 0·001) in both sexes when compared with the UK population mean. Height SDS was similar to the patient’s mid-parental height SDS and the reference population except in the older age group (≥15 years group) who were significantly shorter (*P* = 0·01). There were significant positive correlation of weight SDS (*r* = 0·327; *P* = 0·001) and BMI SDS with age (*r* = 0·368, *P* < 0·001) [Fig. 1(b)], but this trend was not seen with height SDS irrespective of sex. BMI SDS was not related significantly to hydrocortisone dose (*r* = 0·17; *P* = 0·07) but was negatively related to fludrocortisone dose (*r* = −0·38, *P* < 0·001).

**Table 2:** Anthropometric data

Age groups (*n*)	Height SDS Mean (SD)	*P*-value	Delta height SDS Mean (SD)	*P*-value	Weight SDS Mean (SD)	*P*-value	BMI SDS Mean (SD)	*P*-value
0–9·9 years (56)	0·11 (1·66)	0·623	−0·16 (1·5)	0·494	0·27 (1·78)	0·261	0·6 (1·49)	0·004*
10–14·9 years (37)	0·08 (1·68)	0·769	0·07 (1·84)	0·849	1·11 (1·37)	<0·001*	1·43 (1·2)	<0·001*
15 years and above (13)	−0·9 (1·14)	0·014*	−1·03 (1·3)	0·037*	0·69 (1·37)	0·96	1·34 (1·31)	0·003*
All age group (106)	−0·02 (1·63)	0·88	−0·19 (1·6)	0·297	0·61 (1·64)	<0·001*	0·98 (1·42)	<0·001*

Delta Height SDS, Height SDS-Target Ht SDS.

* *P*-value<0·05, significant.

### Hypertension

**Figure 2:**
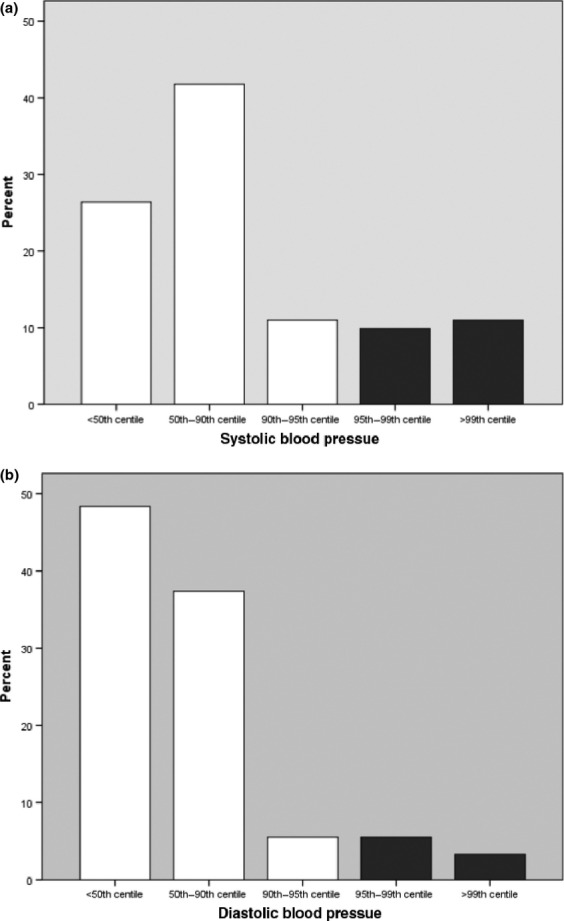
(a) Systolic blood pressure in CAH. (b) Diastolic blood pressure in CAH.

The average SBP in 91 patients with CAH is shown in Fig. 2(a). Nineteen (20·9%) of 91 patients (20% men; 21% women) had systolic hypertension and 10 (11%) patients were noted to have prehypertension. Mean SBP [108 (SD 13·5) mm Hg] and mean SBP SDS 0·76 (SD 1·19) were significantly higher than the reference population (*P* < 0·001). There was a significant negative relationship between SBP SDS and age (*r* = −0·33; *P* = 0·04) but only in men.

The average DBP in 91 subjects is shown in Fig. 2(b). Eight (8·8%) of 91 patients (8·6% men; 8·9% women) had diastolic hypertension with no difference between sexes. Mean DBP [59 (SD 10·8) mm Hg] and mean DBP SDS 0·19 (SD 1·01) were not significantly higher than the reference population (*P* = 0·07). DBP SDS was negatively related to age (*r* = −0·3; *P* = 0·02) in women only. Only 3 (3·3%) patients had both systolic and diastolic hypertension.

### Factors influencing blood pressure

The relationship between systolic and diastolic blood pressure was explored using stepwise multiple linear regression analysis. SBP SDS was predicted only by CA (adjusted *r*^2^ = 0·09; *P* = 0·008), and DBP SDS was predicted by mean plasma cortisol and 9-alpha-fludrocortisone dose (adjusted *r*^2^ = 0·22; *P* = 0·000). Neither SBP SDS nor DBP SDS was significantly related to BMI SDS (*r* = 0·11, *P* = 0·28; *r* = 0·1, *P* = 0·36, respectively).

### Insulin resistance

The difference in the insulin–glucose status between the normal and CAH individuals is shown in Table 3. Fasting blood glucose, fasting plasma insulin and HOMA IR in the CAH subjects were lower than the normal individuals. HOMA IR was positively related to age (*r* = 0·28; *P* = 0·03) but not to BMI SDS (*r* = 0·15; *P* = 0·26).

**Table 3:** Insulin–glucose status between CAH and normal individuals

Age <10 years	Normal (*n* = 56) Mean (SD)	CAH (*n* = 25) Mean (SD)	*P*-value
Fasting glucose	4·6 (0·5)	4·1 (0·6)	<0·001*
Fasting insulin	10 (8)	5 (3·8)	0·002*
HOMA IR	1·3 (0·9)	0·7 (0·4)	0·001*
HOMA%B	132 (66)	104 (43)	0·048*
Age ≥10 years	Normal (*n* = 38) Mean (SD)	CAH (*n* = 32) Mean (SD)	*P*-value
Fasting glucose	4·6 (0·6)	4·4 (0·4)	0·07
Fasting insulin	11·4 (6·3)	8·9 (4·6)	0·07
HOMA IR	1·4 (0·8)	1·1 (0·6)	0·05
HOMA%B	148 (72)	132 (44)	0·288

* *P* = <0·05, significant.

### Hyperlipidaemia

Of 63 subjects, 6 (9·5%) had high plasma triglycerides concentrations and 2 (3%) had high plasma cholesterol concentrations with no differences between sexes. These were not related to age, BMI SDS, blood pressure and hydrocortisone dose.

### Comparison of salt-wasting and simple virilizing types of CAH

There was no significant difference in the age and sex distribution between the SW and SV CAH groups at the time of analysis. Table 4 shows the anthropometric data between the SW and the SV types. The height SDS and weight SDS of SV CAH subjects were significantly higher compared with the SW CAH subjects (*P* < 0·001 for both). There was no significant difference in the prevalence of hypertension, insulin resistance and hyperlipidaemia between these two groups.

**Table 4:** Anthropometric data of SW CAH *vs* SV CAH

	SW CAH (*n* = 78) Mean (SD)	SV CAH (*n* = 28) Mean (SD)	*P*-value
Height SDS	−0·42 (1·43)	1·09 (1·66)	<0·001*
Weight SDS	0·34 (1·65)	1·38 (1·35)	0·003*
BMI SDS	0·87 (1·5)	1·28 (1·14)	0·199

* *P*-value<0·05, significant.

## Discussion

These data demonstrate that obesity and hypertension remain problem areas in children with CAH. Compared with our previous data, there has been a reduction in the numbers who are obese and hypertensive, which probably reflects the lower dosing schedules used since the last publication.^9^ The mean hydrocortisone (13·3 mg/m^2^) and 9-alpha-fludrocortisone (102 μg/m^2^) doses are similar to the recommended doses in the Endocrine Society Practice Guidelines.^8^ Of note, there was a significant negative correlation between the 9-alpha-fludrocortisone dose and the age which reflects our clinical practice not to increase the mineralocorticoid dose on a body size basis once a maximum of 100 micrograms per day has been reached.

Obesity is an independent risk factor for CVD,^21^ and the prevalence in the general population varies and is increasing across the globe.^22^ This has an impact on the prevalence of obesity among CAH subjects and could partly explain the varied prevalence of obesity in different studies along with the disease and treatment factors. The prevalence of obesity in our cohort was 23·6% (33% men; 17·8% women) which is higher than that reported in the Health Survey for England 2010^22^ (17·1% men; 14·8% women). However, men with CAH have a significantly higher risk of obesity than women when compared with the normal population. In other studies in children with CAH, the prevalence of obesity has been reported as 35%^15^ and 16·8%^10^. The mean BMI SDS in our cohort was significantly less (0·98 *vs* 1·57) compared with our previous report a decade ago.^9^ While the prevalence of obesity in general population is increasing, the most likely explanation for this significant decrease in BMI SDS could be due to effect of change in the management of these patients. Interestingly, the mean hydrocortisone dose used in our previous study^9^ was significantly higher (17·5 *vs* 13·3 mg/m^2^), and this may have played an important role in the reduction in the BMI SDS. A more variable prevalence of obesity was reported in adults with CAH, but this may reflect the higher dosing regimens used in the 1970 and 1980s.^11^,^12^,^15^ However, we could not find a significant correlation between the steroid doses and BMI SDS in the current study, and this could be due to small variance in doses used as they are calculated on the basis of body size. BMI is not an accurate estimate of body fat particularly in CAH where androgen effects on muscle mass may be important. Consideration should be given in future studies to the use of dual-energy X-ray absorptiometry to estimate body composition.

The mean height SDS in this cohort was similar to the population mean and the mid-parental SDS in the under 15 year age group indicating optimum growth with current doses of steroids used. However, the mean height SDS was lower in the over 15 year age group and was similar to the adult height reported in the recent meta-analysis.^23^ This might reflect the previous management strategies using higher doses of steroids compared with the current dosage schedule. Although similar trend is seen when compared with target height SDS, we did not have full data on bone age to compare.

The prevalence of hypertension varies widely between studies. The prevalence of systolic (20·9% *vs* 58%) and diastolic hypertension (8·8% *vs* 24%) was significantly less when compared with our previous study but still higher than the population mean.^9^,^24^ This may reflect the lower steroid doses used in our patients compared with our previous study^9^ (mean hydrocortisone dose 13·3 *vs* 17·5 mg/m^2^ and the mean 9-alpha-fludrocortisone dose 102 *vs* 112 μg/m^2^. Interestingly in another study in the paediatric age group, the prevalence of systolic hypertension was only 11% and CAH individuals with normal weight even showed diastolic hypotension.^24^ This might be due to the use of even lower doses of 9-alpha-fludrocortisone compared with our study (mean dose 48 *vs* 102 μg/m^2^). In a recent study^15^, the prevalence of hypertension was even higher than our current study even though the mean hydrocortisone dose used was similar to our study. These differences may relate in part to the dosing as suggested but may also be influenced by the methods for recording blood pressure. We aimed to obtain standardized blood pressure measurements over a 24-h period to avoid, where possible, the possibility of ‘white coat hypertension’. Both SBP SDS and DBP SDS were negatively related to age, and this might be due to reduction in the dose of fludrocortisone, on a body size basis, as age advances. This might also explain why the prevalence of hypertension in the adult CAH population is not as high as that seen in paediatric reports.^12^,^15^ Blood pressure was not related to BMI, in contrast to previous studies^9^,^24^ suggesting that the treatment regimen remains an important factor in generating high BP. The true prevalence of hypertension in our study might be even lower as we used the oscillometric method that tends to overestimate blood pressure by few mmHg compared with sphygmomanometer recordings.^25^

HOMA IR in our CAH cohort was lower than our historical controls, in contrast to previous studies where it was significantly higher.^4^,^15^ This might again be due to the difference in the steroid doses, the taking of samples early morning before the hydrocortisone dose and the difference in the BMI SDS noted between these studies. HOMA IR was also not related to BMI SDS or BP in contrast to the above studies. Although there was a slight increase (9·5%) in the prevalence of hyperlipidaemia in our cohort, we did not have proper controls to assess the significance. Variable reports both in adults and children exist, some showing an increased prevalence^12^,^15^ and others not. 13 Some studies even show that the prevalence of dyslipidaemia is less in CAH patients compared with the general population.^11^,^14^ However, patients with positive family history of dyslipidaemia should have further investigations to rule out familial hypercholesterolaemia.

One of the limitations of our study is that the full data set was not complete in all patients. We also did not evaluate other risk factors such as serum adiponectin, CRP, homocysteine and arterial intima-media thickness.^26^,^27^ However, it is very encouraging to see the decreasing trend in the prevalence of the cardiovascular risk factors in our children with CAH most likely attributed to significant reduction in hydrocortisone and fludrocortisone doses over the last decade. Several individuals are obese, and BMI SDS tends to be higher than average. With the availability of more physiological modalities, such as subcutaneous pumps^28^ or sustained release preparations,^29^ ongoing audit of cardiovascular risk factors is warranted. Assessments such as these should be incorporated into a best practice quality assurance scheme for patients with CAH.

## Supporting Information

Table S1 Annual review programme for children and young people with congenital adrenal hyperplasia.
