# Supplementary material for: Cardiovascular risk factors in children and adolescents with congenital adrenal hyperplasia due to 21-hydroxylase deficiency
Source: Clin Endocrinol (Oxf). 2013 Jul 8;80(4):471–7. doi: 10.1111/cen.12265 (PMC4204515; doi:10.1111/cen.12265)
Supplement: Table S1 — Annual review programme for children and young people with congenital adrenal hyperplasia. [file cen0080-0471-sd1.docx]

**Supplement 1:**

**ANNUAL REVIEW PROGRAMME FOR CHILDREN AND YOUNG PEOPLE WITH CONGENITAL ADRENAL HYPERPLASIA**

|  | **End Point** | **Rationale** | **Measure** |
| --- | --- | --- | --- |
| Short Term | 1. Growth acceleration 2. Weight changes 3. Correct dose for size 4. Blood Pressure   5. Puberty | 1. Assess control 2. Assess dosing 3. Optimise therapy 4. Treatment effects 5. Timing can be altered in CAH | 1. Increase in height velocity ≥ 1SDS needs attention 2. Increase weight gain (i.e. crossing one major centile line in the growth chart) needs attention 3. 1 and 2 above and blood tests 4. Blood Pressure and plot on centile charts 5. Tanner Staging |
| Medium Term | 1. Bone maturation 2. Pubertal Status 3. Hydrocortisone Dose 4. Fludrocortisone Dose 5. Ovary and testes health 6. Metabolic Status | 1. Rate of skeletal maturation 2. Early puberty or rapid progression 3. Optimise therapy 4. Avoid high blood pressure endocrinopathy 5. PCOS and Adrenal Rests 6. Insulin insensitivity and Dyslipidemia | 1. Yearly Bone age 2. Tanner staging 3. Cortisol and 17OHP profiles over 24 hour period 4. Plasma Renin Activity 5. Pelvic ultrasound for girls. Careful examination and testicular ultrasound for boys 6. Fasting glucose, insulin and lipids |
| Long Term | 1. Growth 2. Bone mineralization 3. Fertility 4. Cardiovascular risk | 1. Outcome 2. CAH/treatment effect on bone 3. Effect of CAH 4. CAH/treatment effect | 1. Final height within target height of parents 2. DEXA Scan 3. Check for regular menstrual cycle (girls) and adrenal rests in testes in boys 4. Fasting glucose and insulin, blood pressure, fasting lipids |
